# Sand flies (Diptera: Psychodidae) in eight Balkan countries: historical review and region-wide entomological survey

**DOI:** 10.1186/s13071-020-04448-w

**Published:** 2020-11-11

**Authors:** Vit Dvorak, Ozge Erisoz Kasap, Vladimir Ivovic, Ognyan Mikov, Jovana Stefanovska, Franjo Martinkovic, Jasmin Omeragic, Igor Pajovic, Devrim Baymak, Gizem Oguz, Kristyna Hlavackova, Marketa Gresova, Filiz Gunay, Slavica Vaselek, Nazli Ayhan, Tereza Lestinova, Aleksandar Cvetkovikj, Darinka Klaric Soldo, Ivelina Katerinova, Simona Tchakarova, Ayda Yılmaz, Begum Karaoglu, Jose Risueno Iranzo, Perparim Kadriaj, Enkelejda Velo, Yusuf Ozbel, Dusan Petric, Petr Volf, Bulent Alten

**Affiliations:** 1 Faculty of Science, Department of Parasitology, Charles University, Prague, Czech Republic; 2 Department of Biology, Ecology Section, Faculty of Science, VERG Laboratories, Hacettepe University, Ankara, Turkey; 3 Science and Research Centre, University of Primorska, Koper-Capodistra, Slovenia; 4 Department of Parasitology and Tropical Medicine, National Centre of Infectious and Parasitic Diseases, Sofia, Bulgaria; 5 Department of Parasitology and Parasitic Diseases, Faculty of Veterinary Medicine-Skopje, Ss. Cyril and Methodius University in Skopje, Skopje, North Macedonia; 6 Department for Parasitology and Parasitic Diseases with Clinics, Faculty of Veterinary Medicine, University of Zagreb, Zagreb, Croatia; 7 Veterinary Faculty, Department of Parasitology and Invasive Diseases of Animals, University of Sarajevo, Sarajevo, Bosnia and Herzegovina; 8 Biotechnical Faculty, University of Montenegro, Podgorica, Montenegro; 9 National Institute of Public Health, Pristina, Kosovo; 10 Laboratory for Medical and Veterinary Entomology, Department of Plant and Environmental Protection, Faculty of Agriculture, University of Novi Sad, Novi Sad, Serbia; 11 Unite des Virus Emergents (UVE: Aix Marseille Univ IRD 190, INSERM 1207 IHU Mediterranee Infection), 13005 Marseille, France; 12 EA7310, Laboratoire de Virologie, Université de Corse-Inserm, 20250 Corte, France; 13 National Diagnostic and Research Veterinary Medical Institute, Sofia, Bulgaria; 14 Departamento de Sanidad Animal, Facultad de Veterinaria, Regional Campus of International Excellence “Campus Mare Nostrum”, Universidad de Murcia, Murcia, Spain; 15 Department of Epidemiology and Control of Infectious Diseases Department, Institute of Public Health, Tirana, Albania; 16 Department of Parasitology, Faculty of Medicine, Ege University, İzmir, Turkey

**Keywords:** Phlebotomine, Sand fly, Balkans, Species, Morphology

## Abstract

**Background:**

Sand flies (Diptera: Psychodidae) are medically important vectors of human and veterinary disease-causing agents. Among these, the genus *Leishmania* (Kinetoplastida: Trypanosomatidae), and phleboviruses are of utmost importance. Despite such significance, updated information about sand fly fauna is missing for Balkan countries where both sand flies and autochtonous leishmaniases are historically present and recently re-emerging. Therefore, a review of historical data on sand fly species composition and distribution in the region was followed by a large-scale entomological survey in eight Balkan countries to provide a recent update on local sand fly fauna.

**Methods:**

The literature search involved the period 1910–2019. The entomological survey was conducted at 1189 sampling stations in eight countries (Bulgaria, Bosnia and Herzegovina, Croatia, Kosovo, Montenegro, North Macedonia, Serbia and Slovenia), covering 49 settlements and 358 sampling sites between June and October in the years 2014 and 2016, accumulating 130 sampling days. We performed a total of 1189 trapping nights at these stations using two types of traps (light and CO_2_ attraction traps) in each location. Sampling was performed with a minimal duration of 6 (Montenegro) and a maximal of 47 days (Serbia) between 0–1000 m.a.s.l. Collected sand flies were morphologically identified.

**Results:**

In total, 8490 sand fly specimens were collected. Morphological identification showed presence of 14 species belonging to genera *Phlebotomus* and *Sergentomyia.* Historical data were critically reviewed and updated with our recent findings. Six species were identified in Bosnia and Herzegovina (2 new records), 5 in Montenegro (2 new records), 5 in Croatia (2 new records), 9 in Bulgaria (5 new records), 11 in North Macedonia (1 new record), 10 in Serbia (no new records), 9 in Kosovo (3 new records) and 4 in Slovenia (no new records).

**Conclusions:**

This study presents results of the first integrated sand fly fauna survey of such scale for the Balkan region, providing first data on sand fly populations for four countries in the study area and presenting new species records for six countries and updated species lists for all surveyed countries. Our findings demonstrate presence of proven and suspected vectors of several *Leishmania* species.

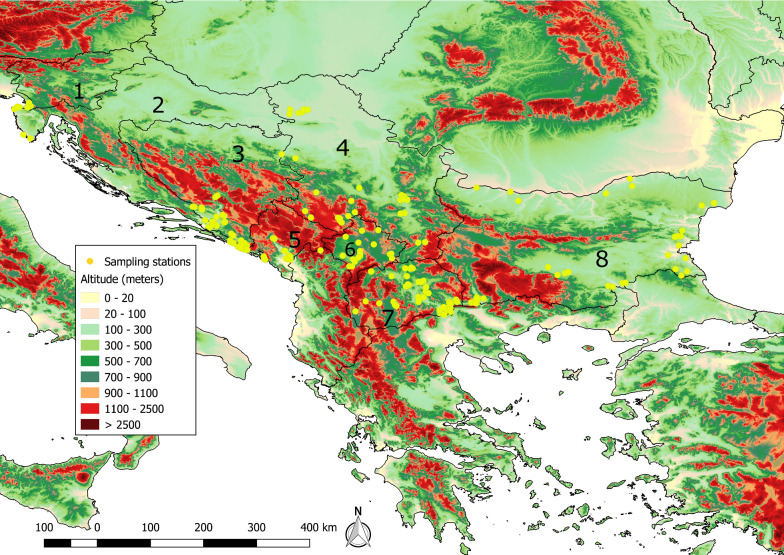

## Background

Phlebotomine sand flies (Diptera: Psychodidae) are medically important as the vectors of *Leishmania* spp. and arboviruses that threaten human and animal health. Within the subfamily Phlebotominae, over 900 species were described so far, of which at least 100 species of the genera *Phlebotomus* and *Lutzomyia* are suspected or proven vectors of *Leishmania* spp. in the Old and New World [1]. Leishmaniases, diseases caused by parasitic protozoans of the genus *Leishmania* (Kinetoplastida: Trypanosomatidae), infect approximately two million people annualy, with Latin America, East Africa, Indian subcontinent, the Middle East and the Mediterranean Basin being among the most affected regions [2]. In addition to this burden, sand flies also transmit several medicaly important viruses like sand fly fever Sicilian and Toscana virus of the genus *Phlebovirus* and Chandipura and Isfaham viruses of the genus *Vesiculorivus* [1]. Recent studies in the Mediterranean Basin have shown that the diversity and prevalence of these viruses is much higher than previously known [3–5], further highlighting the importance of updated knowledge of sand fly fauna of the region.

Knowledge of vector species distribution is crucial for the assessment of vector-borne disease risk. This was prioritized by European Centre for Disease Prevention and Control (ECDC) and European Food Safety Authority (EFSA), both agencies jointly funding in the periods 2010–2014 and 2014–2018, respectively, two consecutive projects named VborNet and VectorNet. The enormous effort to extract presence/absence data from published literature during the VborNet project resulted in a large, validated, high-quality dataset of European sand flies. Gap analysis performed to detect possible missing data and to determine the areas with less or no information of sand fly species in the Western Palearctic and particularly in Europe revealed several understudied regions including the Balkans. The apparent lack of updated information about sand fly fauna, despite records of sand fly-borne diseases in the past as well as recently, is understandable in the light of political and social upheavals in this region during the last three decades. Neighboring Mediterranean countries in the west are well known to be endemic for leishmaniasis caused by *Leishmania infantum* but their sand fly fauna presumably partially differs in species composition [1]. The Balkan region was therefore chosen as a target of a thorough entomological field survey both for a striking lack of updated and validated data on sand fly vectors and for its importance for vector/pathogen transmission between western European countries and Anatolia, Caucasus, Middle East countries. The region comprises 13 countries namely Albania, Bosnia and Herzegovina, Bulgaria, Croatia, Greece, Kosovo, Moldova, Montenegro, North Macedonia, Romania, Serbia, Slovenia and Turkey (Eastern Thrace). Of these, we primarily focused on the former Yugoslavian countries and Bulgaria.

The present study presents results of entomological collections of sand flies in eight countries (Bulgaria, Bosnia and Herzegovina, Croatia, Kosovo, Montenegro, North Macedonia, Serbia and Slovenia) through extensive field studies performed between 2014 and 2016 during the VectorNet Project. We provide the first checklists of sand fly species for several surveyed countries and updated checklists for the remaining countries. We compare these checklists with the historical records reviewed from previously published studies with the aim to critically assess the past and current species composition of sand fly fauna in this important yet understudied region.

## Methods

### Literature search and data extraction

The literature search follows the Prisma Journal Publishing protocol workflow [6]. PubMed, Web of Science, Ovid Medline, CAB Direct, Google Scholar databases and web searches were screened from 1910 to 2019. Full text articles, reports, theses, congress presentations, book chapters in English language containing information on phlebotomine sand flies from the Balkan region were selected. Other articles, including those published in other languages that contain valuable information were also included in the data set. Some data on phlebotomine sand flies also comes from direct consultations with experts and their in-house unpublished databases.

The following search string was used. Terms in title: [(phlebotomine OR sandflies OR sand flies) AND in all fields: (*Phlebotomus* OR *Sergentomyia*) AND in all fields: (*Phlebotomus* OR *Paraphlebotomus* OR *Larroussius* OR *Adlerius* OR *Transphlebotomus*) AND in all fields: (species name) AND in all fields: (distribution OR presence OR occurrence OR report OR spread OR dispers OR detect) AND in all fields:(“former Yugoslavia” OR Yugoslavia OR “Balkan region” OR “Mediterranean area” OR Europe OR Balkans OR “Thrace Region” OR Trakia OR Trakya OR Bulgaria OR Macedonia OR “FYROM” OR North Macedonia OR Serbia OR Kosovo OR Montenegro OR Croatia OR Slovenia OR Bosnia and Herzegovina OR Herzegovina) AND in all fields: (leishmaniasis OR “cutaneous leishmaniasis” OR “CL” OR “visceral leishmaniasis” OR “VL” “canine leishmaniasis ” OR “CanL” OR “phlebovirus”) AND in all fields: (“neglected tropical diseases” OR “vector-borne diseases”) AND in all fields: (“vector control”)].

Historical data extraction study revealed that studies on sand fly fauna in the Balkans mostly coincide with the times of leishmaniasis and/or phlebovirus epidemics that started in the region and were then taken under control. The research was suspended due to the Second World War between 1940–1945. Therefore, the results were evaluated in four time periods: (i) 1910–1940; (ii) 1945–1955; (iii) 1967–1990; (iv) 1990 to present time.

### The study area and sand fly sampling

**Fig. 1:**
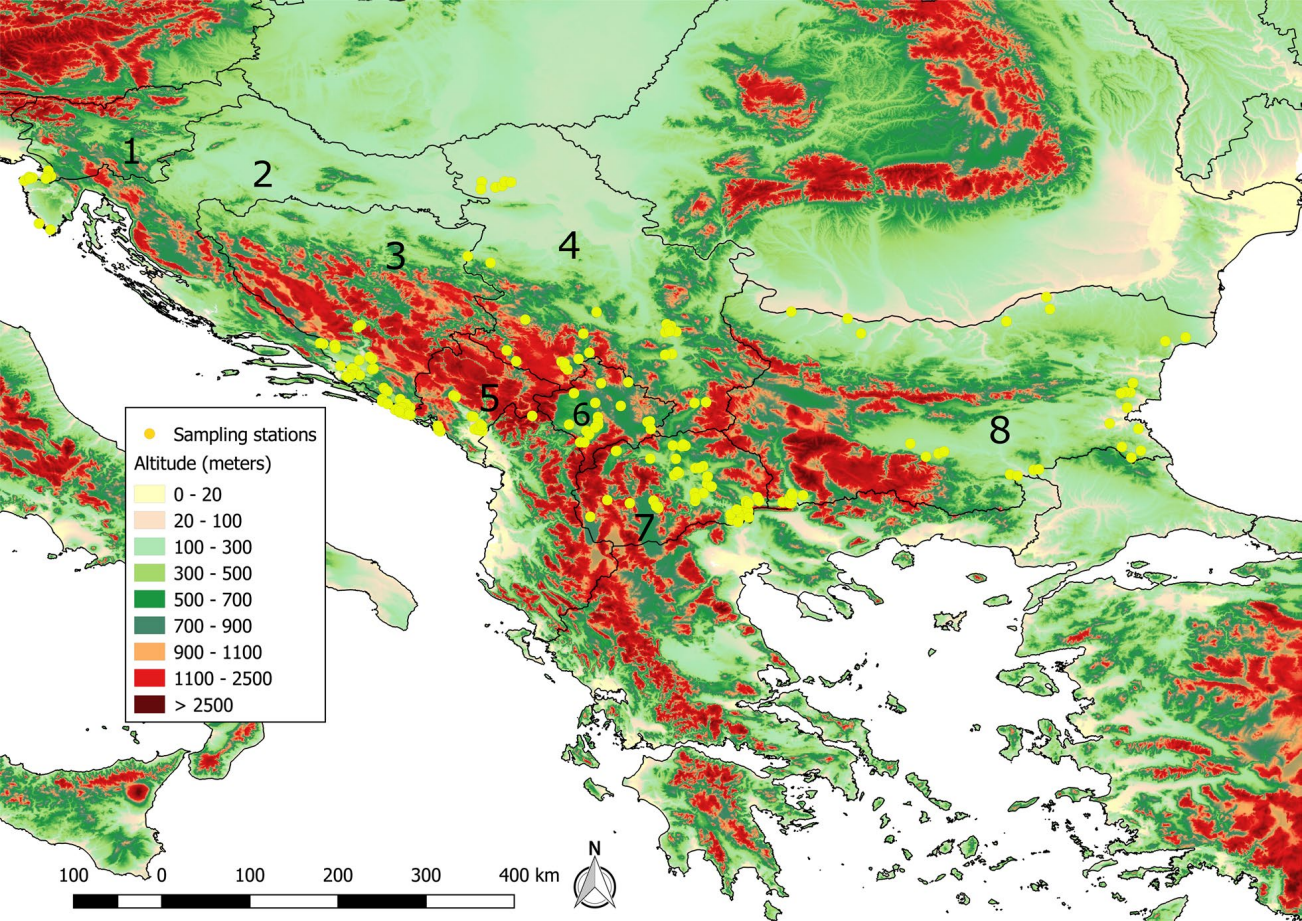
Map showing the sand fly sampling locations (yellow dots) in the studied countries. The base map of each country and the elevation data was acquired from http://www.diva-gis.org/gdata. 1: Slovenia; 2: Croatia; 3: Bosnia & Herzegovina; 4: Serbia; 5: Montenegro; 6: Kosovo; 7: North Macedonia; 8: Bulgaria

Eight countries were selected for sand fly sampling in the Balkans as a result of the extensive gap analysis during the VborNet project: Bosnia and Herzegovina, Croatia, Kosovo, Montenegro, North Macedonia, Serbia, Slovenia (former Yugoslavian countries) and Bulgaria (Fig. 1). Sampling was done during the active sand fly season, typically from June to October, between 2014 and 2016 (Table 1). Some sampling sites were chosen based on previously published data on sand fly fauna and/or leishmaniasis cases or the data from local veterinarians who participated in the field collections, with regards to presence of new cases of canine and human leishmaniasis. The remaining stations were chosen according to suitable altitudes, habitats, climatic parameters, presence of hosts and potential breeding sites as known from previous field studies.

**Table 1:** Trapping effort according to countries in the study area

Country	Bulgaria	North Macedonia	Serbia	Kosovo	Bosnia and Herzegovina	Montenegro	Croatia	Slovenia	Total
Trapping									
No. of sampling locations	63	91	74	51	32	18	23	6	358
No. of sampling stations	246	230	275	118	126	51	95	48	1189
No. of trap/night	246	230	275	118	126	51	95	48	1189
No. of settlements	9	9	12	7	3	6	2	1	49
No. of sampling days	14	27	47	10	7	6	12	7	130
Sampling month of the year	8	8, 9	6, 7, 8, 9	7, 9	7	7	7, 8	6, 7	6–10
Identification									
No. of specimens	679	2309	199	292	2496	246	1739	530	8490
No. of validated specimens (%)^a^	83 (12.2)	237 (10.3)	199 (100)	33 (11.3)	272 (10.9)	38 (15.4)	180 (10.3)	67 (11.3)	1109 (13.1)

^a^%, proportion of validated specimens

As summarized in Table 1, 1189 sampling stations were identified in eight targeted countries, in a total of 49 settlements and 358 sampling sites during 130 days. We performed a total of 1189 trapping nights at these stations using 2–6 light and CO_2_ attraction traps in each location as described below. Taking into consideration the topography of the surveyed regions, sampling was performed with a minimal duration of 6 (Montenegro) and a maximal of 47 (Serbia) days between 0–1000 m.a.s.l. In Serbia, sand flies were sampled repeatedly in two consecutive years as initial catch was negatively affected by unfavourable weather conditions.

In order to maximize the yield of sand fly samples, samplings followed a standardized protocol (“The Field Sampling Standard Protocol of VectorNet Project”) and previously published standard methods [7, 8]. Since this was a presence/absence study, traps were positioned only once per site. Minimum of 2 and maximum of 6 traps were placed per each site including Centre for Disease Control (CDC) light traps (John W. Hock Company, model 512, Gainesville, Florida, USA) and CDC light traps with carbon dioxide (dry ice) (GENICCO S.r.l., Milan, Italy) in all countries. Sand fly surveys were usually performed at protected private properties which are mostly farms with presence of various domestic mammals and poultry. Traps were placed both outside or inside the houses and animal shelters when possible. However, to detect the maximum sand fly species diversity, traps were placed also at other highly urban, semiurban or sylvatic sites. The traps were placed approximately 1–1.5 m above the ground, and run approximately 2 h before the sunset and switched off 2 h after sunrise. Being retrieved from the traps, the specimens were immediately placed in 70% ethanol for later morphological identification.

In order to better understand the distribution and occurrence of different species, we evaluated each country according to frequency of occurrence (%) and proportion of total samples belonging to species x (%). In the countries where sand fly monitoring was performed, frequency of occurrence of different sand fly species is expressed as a ratio of positive traps in all trapping stations. The formulae and their rationale in the present study are summarized as follows: Frequency of occurrence: s_x_/n_t_*100 (where s_x_ is the species trapped and n_t_ is the number of positive traps); Proportion: n_x_/N*100 (where n_x_ is the number of individuals belonging to species x and N is the total number of sampled individuals).

### Species identification

All four VectorNet-affiliated laboratories in Turkey (Hacettepe University-VERG), Czech Republic (Charles University-CUNI), Slovenia (University Primorska-UPBIO) and Serbia (University of Novi Sad, Medical Entomology Laboratory) were responsible for species identification. When performed by each team, a proportion (10%) of the samples was identified by other consortium laboratories for quality check and validation.

The head and the terminal 2–3 abdominal segments of each specimen were dissected, cleared in boiling Marc-André solution and mounted in a drop of Berlese or CMCP (Polysciences, Warrington, USA) for morphological identification using the available keys [9–11] and the expertise of members of the project. The head was mounted dorsally, male genitalia laterally and female spermathecae were dissected before mounting. The thorax and the rest of the abdomen of each specimen were stored in 70% or 96% ethanol for future application of molecular methods DNA barcoding [12–15] and MALDI-TOF protein profiling [16, 17]. Results of these analyses will be published separately.

## Results

### Historical data review on the sand fly fauna of the Balkan region

Detailed literature search and analysis of the data divided the research on sand fly fauna in former Yugoslavia and Bulgaria into four main periods: (i) 1910–1940; (ii) 1945–1955; (iii) 1967–1990; (iv) 1990 to present time (Additional file 1: Table S1, Table 2).

**Table 2:** Checklist of sand fly records in the study area

Sand fly species	Bulgaria		North Macedonia		Serbia		Kosovo		B&H		Montenegro		Croatia		Slovenia	
	Pres	Ref	Pres	Ref	Pres	Ref	Pres	Ref	Pres	Ref	Pres	Ref	Pres	Ref	Pres	Ref
Subgenus *Phlebotomus* Rondani & Berte, 1840																
*Phlebotomus papatasi* (Scopoli, 1786)	x	[42, 44, 45, 47–55, 78, 79]	x	[21, 25, 32, 49, 71, 77, 95]	x	[19, 21, 24, 26, 27, 29, 30, 32, 38, 95]	x	[25, 32]	x	[30, 32, 71, 95]		[32–35, 71, 73, 75, 95]	x	[21, 23, 30, 32, 71, 73–75, 83–85, 95]	x	[30, 37, 95]
Subgenus *Paraphlebotomus* Theodor, 1948																
*Phlebotomus alexandri* Sinton, 1928	x		x		x	[27]	x									
*Phlebotomus sergenti* Parrot, 1917	x	[42, 44, 47, 78]	x	[21, 24, 25, 32, 71, 95]	x	[21, 25, 27, 29, 32, 71, 95]	<	[25, 32]	<	[32, 71, 95]	<	[32, 33, 71, 95]	<	[21, 32, 71, 84, 95]	<	[32, 95]
Subgenus *Larroussius* Nitzulescu, 1931																
*Phlebotomus kandelakii* Schurenkova, 1929	x										<	[35]				
*Phlebotomus major* Annandali, 1910^a^			<	[22, 32, 71, 77]	<	[19, 24, 29, 30, 32, 71]	<	[25, 26, 32]	<	[30, 32, 71]	<	[32, 33, 71, 72]	<	[22, 30, 32, 71]	<	[32]
*Phlebotomus neglectus* Tonnoir, 1921	x		x	[1, 21, 22, 25, 95]	x	[21, 24, 27, 28, 38, 95]	x	[1]	x		x	[1, 28, 35, 74, 75, 95]	x	[1, 21, 23, 28, 36, 75, 83, 85, 95]	x	[1, 37, 95]
*Phlebotomus pedifer* Lewis, Mutinga & Ashford, 1972^a^											<	[32]				
*Phlebotomus perfiliewi* Parrot, 1939	x		x	[1, 21, 22, 25, 29, 32, 71, 72, 77, 95]	x	[19, 21, 24, 30, 32, 38, 71, 76, 95]	x	[1, 25, 32]	<	[30, 32, 71, 95]	<	[1, 28, 32, 34, 35, 71, 74, 75, 95]	<	[1, 21, 23, 28, 30, 32, 36, 71, 73, 75, 84, 95]	<	[1, 32, 95]
*Phlebotoms perniciosus* Newstead, 1911	<	[42, 45, 47]	<	[21, 22, 32]	<	[21, 28, 32]	<	[32]	<	[32]	<	[28, 32]	x	[21, 22, 28, 32, 84]	x	[32, 37]
*Phlebotomus tobbi* Adler, Theodor& Lourie, 1930	x	[44–46]	x	[21, 25, 77, 95]	x	[21, 24, 28, 30, 76, 95]	x	[25]	x	[30, 95]	x	[28, 33–36, 73–75, 95]	x	[21, 23, 28, 30, 73–75, 83–85, 95]	<	[95]
Subgenus *Adlerius* Nitzulescu, 1931																
*Phlebotomus balcanicus* Theodor, 1958	<	[43, 45, 78]	x	[21, 31, 72]	x	[21, 24, 27–29, 31]	x	[31, 72]	<	[31]	x		<	[21, 28, 31]	<	[31]
*Phlebotomus chinensis* Newstead, 1916^a^	<	[47, 79]	<	[77]												
*Phlebotomus simici* Nitzulescu, 1931	<	[31]	x	[21, 25, 32, 77]	x	[19, 21, 24–30, 32, 76]	x	[25, 32]	x	[30, 32]	x		<	[21, 28, 30, 32]	<	[32]
Subgenus *Transphlebotomus* Artemiev, 1984																
*Phlebotomus mascittii* Grassi, 1908			<	[32]	x	[27, 32, 38]	x		x		<	[32]	<	[32, 36]	x	[32, 37]
Subgenus *Sergentomyia* França & Parrot, 1920																
*Sergentomyia dentata* Sinton, 1933	x		x	[21, 25, 26, 32, 77]	<	[21, 28, 32]	<	[32]	<	[32]	<	[26, 28, 32]	<	[21, 26, 28, 32]	<	[32]
*Sergentomyia minuta* (Rondani, 1843)	x	[42, 47, 53–55]	x	[21, 25, 26, 32]	x	[21, 28, 30, 32]	x		x	[30, 32]	x	[26, 28, 32–35, 73–75]	x	[21, 28, 30, 32, 36, 73–75, 83–85]	<	[32, 37]

^a^Misidentification and/or species complex

*Abbreviations*: Pres, presence; Ref, reference; B&H, Bosnia and Herzegovina

*Key*: x, the first record of certain species in the study area; x, species recorded in previous studies and the present study; <, species recorded by previous authors

*Note*: The subgenera and species are classified in accordance with Theodor [9], Artemiev [10] and Lewis [11]

As far as we can reach, the research began with the publication of a febrile disease detected in the city of Trebinje in Bosnia and Herzegovina in 1886. Czech Alois Pick, an Austrian-Hungarian military doctor, changed the name of the disease, previously known as “gastroenteritis endemica” to “dog disease”, and identified “sand fly fever” for the first time without knowing that it was transmitted by sand flies [18]. The disease was seen among immigrants in many cities of Bosnia and Herzegovina triggering further studies. Before the World War II, the papatasi fever in the former Yugoslavia was known mainly in Herzegovina, Dalmatia, Montenegro and especially in Macedonia, where its prevalence coincided with that of *Phlebotomus papatasi* [18]. In Serbia, it was first detected after World War II, later becoming epidemic in the entire former Yugoslavia [19, 20].

In the same period, the first case of visceral leishmaniasis (VL) in former Yugoslavia was recorded in 1911 in Trieste in a man from Zadar and occasional cases were reported in Macedonia, Herzegovina, Dalmatia and Montenegro between the two World Wars, while several major epidemics occurred in Serbia. Research was not specifically focused on sand fly fauna during this period [21] but six species were reported from countries that would later make up former Yugoslavia until the beginning of the World War II: *P. neglectus*; *P. perfiliewi*; *P. papatasi*; *P. tobbi*; *P. major*; and *P. perniciosus* [22, 23].

Immediately after the World War II (the years in second period), sand fly fever was officially considered to have an epidemic characteristic, while cases of cutaneous leishmaniasis (CL) and VL from many regions of former Yugoslavia began to increase very quickly [21]. The first VL case was detected between 1944 and 1945 in Dobrič, Serbia [24] and later it occurred with increasing mortality rate in many regions of the former Yugoslavia, mostly in Serbia but with sporadic cases reported from across the country in 1956, prompting faunistic studies on sand flies between 1947 and 1953, particularly in areas where the disease was endemic. Most studies focused on Dobrič in Serbia, the Bar region in Montenegro, the coastal regions of Croatia and the Adriatic Sea islands; however, Kosovo, Macedonia, Vojvodina region in northern Serbia and the central region of Serbia were also surveyed. Between 1947 and 1957, nine species (*P. simici*, *P. neglectus*, *P. perfiliewi*, *P. papatasi*, *P. sergenti*, *P. tobbi*, *P. major*, *Sergentomyia dentata* and *S. minuta*) were reported from different regions of the country and *P. major* has attributed a crucial role in the epidemiology of VL in former Yugoslavia [25, 26]. Compared with the first period, there were three new species identified in the former Yugoslavia. [27].

In the third period (1967–1990), intensive research focused on sand fly species in Dobrič, the most imperilled endemic focus of VL in the previous period that provided 8 cases in children in 1968 [21] Faunistic studies were generally carried out in urban and rural settlements and animal stables in residential areas and in various microhabitats outside the settlements such as rodent burrows, cracks of stone walls on road sides, forests, corn fields, vineyards and areas where fertilizers had been applied [28]. *Phlebotomus balcanicus* was identified for the first time in addition to the 9 species previously recorded in the second period [29], raising the number of sand fly species in the former Yugoslavia to 10.

In the fourth period (from 1990 to the present), faunistic studies and publications on sand flies are relatively limited, partly due to political instability and the unfavourable economical situation followed by civil war. Mostly catalogues, reviews and monographs based on older literature were published [1, 2, 28, 30–32]. To the best of our knowledge, there are at least seven research articles publishing results obtained by field studies [27, 33–38]. Among them, Ivovic et al. [34] in Bar region in Montenegro and Bosnic et al. [36] in Dalmatia region added *P. kandelakii* (unconfirmed) and *P. mascittii*, respectively, to the species list of former Yugoslavia. Besides these species, *P. perfiliewi* was recorded for the first time by Ivovic et al. [26] in the Bar district of Montenegro. The first sand fly fauna of the Istrian Peninsula region of Slovenia identified five species including *P. neglectus*, *P. perniciosus*, *P. papatasi*, *P. mascittii* and *S. minuta* in the region [37] and *P. mascitti* was also recorded in Croatia [36]. Recent occurrence of new leishmaniasis cases in Serbia provided new information on sand fly fauna in 2017 and in 2019 [27, 38], reporting a total of 10 species (*P. papatasi*, *P. perfiliewi*, *P. tobbi*, *P. neglectus*, *P. sergenti*, *P. simici*, *P. balcanicus*, *P. mascittii*, *P. alexandri* and *S. minuta*). The first record of *P. alexandri* increased the total number of sand fly species reported since 1900 in the former Yugoslavia countries to 12.

In Bulgaria, the first imported VL case was reported in 1921 and the first domestic case occurred in 1937 [39]. Until 1953, a total of 57 autochthonous cases were reported in the country, 50 of which were children. All cases were recorded sporadically, mostly in the south of the country [40, 41]. During the following 35 years, all cases recorded in the country were also sporadic. While all cases of leishmaniasis are followed clinically in the country, studies on sand fly fauna are strikingly limited. In total, the presence of seven *Phlebotomus* species has been reported in Bulgaria in the past: *P. papatasi*; *P. sergenti*; *P. pernicious*; *P. balcanicus*; *P. simici*; *P. tobbi*; and *S. minuta* [31, 41–55].

### Entomological survey

In the field study done during three sand fly seasons (2014–2016), a total of 8490 sand fly specimens were collected and morphologically identified (Table 3). Within these, identification of 1109 (13.1%) specimens was checked and confirmed (100%) by four VectorNet-affliated laboratories. In total, 14 species belonging to two genera (*Phlebotomus* and *Sergentomyia*) and six subgenera (*Phlebotomus*, *Paraphlebotomus*, *Larroussius*, *Adlerius*, *Transphlebotomus* and *Sergentomyia*) were recorded (Tables 2, 3). The most abundant species was *P. neglectus*, accounting for 62.03% of all the flies collected. *Phlebotomus neglectus* was also recorded in all surveyed countries. The second most abundant species in the study area, *P. perfiliewi* (18.66%), was recorded in Bulgaria, North Macedonia, Serbia and Kosovo, and the third species *P. tobbi* (11.59%) in all countries except Slovenia.

**Table 3:** Sand fly species, number of specimens caught and new species records in the countries of study area

Species	Bulgaria	North Macedonia	Serbia	Kosovo	Bosnia and Herzegovina	Montenegro	Croatia	Slovenia	Total	%^b^
*Phlebotomus (Larroussius) neglectus* Tonnoir, 1921	201^a^	517	113	222	2241^a^	181	1365	426	5266	62.03
*Phlebotomus (Larroussius) perfiliewi* Parrot, 1939 (*s.l.*)	132^a^	1441	9	2	0	0	0	0	1584	18.66
*Phlebotomus* (*Larroussius*) *tobbi* Adler, Theodor & Lourie, 1930	246	254	22	29	222	45	166	0	984	11.59
*Phlebotomus* (*Phlebotomus*) *papatasi* (Scopoli, 1786)	73	24	25	3	11	0	3	3	142	1.67
*Phlebotomus* (*Larroussius*) *perniciosus* Newstead, 1911	0	0	0	0	0	0	23	77	100	1.18
*Phlebotomus* (*Adlerius*) *simici* Nitzulescu, 1931	0	22	8	14	13	5^a^	0	0	62	0.73
*Phlebotomus* (*Transphlebotomus*) *mascittii* Grassi, 1908	0	0	5	4^a^	3^a^	0	0	24	36	0.42
*Phlebotomus* (*Adlerius*) *balcanicus* Theodor, 1958	0	4	12	14	0	2^a^	0	0	32	0.38
*Phlebotomus* (*Paraphlebotomus*) *sergenti* Parrot, 1917	7	4	3	0	0	0	0	0	14	0.16
*Phlebotomus* (*Larroussius*) *kandelakii* Schurenkova, 1929	12^a^	0	0	0	0	0	0	0	12	0.14
*Phlebotomus* (*Paraphlebotomus*) *alexandri* Sinton, 1928	1^a^	2^a^	1	3^a^	0	0	0	0	7	0.08
*Paraphlebotomus* sp.	0	2	0	0	0	0	0	0	2	0.02
*Sergentomyia* (*Sergentomyia*) *minuta* (Rondani, 1843)	4	28	1	1^a^	6	13	182	0	235	2.77
*Sergentomyia* (*Sergentomyia*) *dentata* Sinton, 1933	3^a^	11	0	0	0	0	0	0	14	0.16
Total	679	2309	199	292	2496	246	1739	530	8490	
No. of species	9	11	10	9	6	5	5	4	14	
Density^c^	2.76	10.04	0.72	2.47	19.80	4.82	18.3	11.04	7.14	

^a^The first record(s) in the country

^b^nx/N*100, nx: number of individuals belonging to species x, N: total number of sampled individuals

^c^D, number of specimens/number of trap-nights

Following the methods described in Pudar et al. [56], data were grouped into three categories. Categories were constructed by using the relative abundance (proportion) and frequency of each sand fly species collected in each country during the study period without any ecological considerations: (i) Bulgaria, North Macedonia and Serbia, Kosovo; (ii) Bosnia and Herzegovina; and (iii) Montenegro, Croatia and Slovenia. In the first category, the species with the highest proportion were determined as *P. perfiliewi* (45.53%) followed by *P. neglectus* (30.26%) and *P. tobbi* (15.83%). The less abundant species were *P. papatasi*, *P. simici*, *S. minuta* and *P. balcanicus*. The remaining species were caught at low numbers. Due to the fact that *P. perfiliewi* was the most abundant species (62.40%; *n* = 1441) (Table 3) in a relatively limited area (center and North-West of the country) in North Macedonia, it has been identified as the dominant species for this category. In contrast, frequency of occurrence calculations showed that this species is not the most frequent species in these countries. *Phlebotomus neglectus* caught at 52.47% of sampling stations (*n* = 456/869) was the most frequent species in this category followed by *P. tobbi* (38.55%; *n* = 335), *P. perfiliewi* (18.29%; *n* = 159), *P. papatasi* (9.24%; *n* = 80), *P. simici* (2.61%; *n* = 23), *S. minuta* (1.84%; *n* = 16) and *P. balcanicus* (1.38%; *n* = 12). The remaining species were very rare in the area. *Phlebotomus kandelakii* (*s.l*.) was only recorded in Bulgaria. The highest species richness (9–11 species) was found in the countries of this category.

In Bosnia and Herzegovina (second category), *P. neglectus* was the most abundant species with the proportion of 89.78% among five other species collected in the country. The second most abundant species was *P. tobbi* (8.89%) in this category. The remaining four species, *P. simici*, *P. papatasi*, *S. minuta* and *P. mascittii*, were represented at very low numbers. *Phlebotomus neglectus*, again, was distinctively the most frequent species recorded at 95.23% (*n* = 120/126 total stations) of sampling stations, followed by *P. papatasi* (43.65%; *n* = 55). All other four species were collected at only less than 10% (9.52%) of stations (*n* = 12).

In the third category that includes Montenegro, Croatia and Slovenia, the species richness was found to be very low (4–5 species). *Phlebotomus neglectus* was recorded as the most abundant species on the coastline of the study area (78.40%) and particularly in Croatia (78.50%) in total samples (Table 3), followed by *P. tobbi* (8.38 %) and *S. minuta* (7.75%). *Phlebotomus neglectus* caught at 95.36% of sampling stations (*n* =185/194 total stations) was also the most frequent species in coastal part of the study area, followed by *P. tobbi* (25.77%; *n* = 50), *S. minuta* (23.19%; *n* = 45), *P. perniciosus* (20.61%; *n* = 40), while the remaining species were found to be rare. In contrast to other categories, *S. minuta* was collected with the highest number (*n* = 13 in Montenegro, *n* = 182 in Croatia) in the coastal area. The highest collection of this species was made in Croatia (Table 3).

In total, varying numbers of species were recorded in surveyed countries during this study (Tables 2, 3) including new records of several species in some of them: *P. alexandri* in North Macedonia; *P. alexandri*, *P. neglectus*, *P. kandelakii*, *P. perfiliewi* (*s.l.*), *S. dentata* in Bulgaria; *P. mascittii*, *P. alexandri*, *S. minuta* in Kosovo; *P. neglectus* and *P. mascittii* in Bosnia and Herzegovina; and *P. simici* and *P. balcanicus* in Montenegro.

## Discussion

Sand fly fauna of the Balkans remains least known within the Mediterranean basin and was understudied for several decades, despite the role of sand flies as vectors *Leishmania* spp. and other pathogens. Genetic diversity and population structures of *L. infantum* assessed by microsatellite analysis in southeastern Europe comprising Croatia, Albania, Greece, Aegean Islands, Bulgaria and Turkey showed that the MON-1 strain is predominant in the southeastern Mediterranean region, causing canine leishmaniasis (CanL) in dogs in Albania, Bulgaria and Croatia [57]. Recent data suggests that leishmaniasis due to *L. infantum* is now a re-emerging disease in the Balkans after several decades of relatively few reported cases. A clinical study in Bulgaria in 2013 revealed that 59 out of 120 human patients showed common VL symptoms [58], and VL tests of 166 dogs in the same country also revealed three cases of CanL in 2015 [46]. In a study in the Tuzla region (Bosnia and Herzegovina) in 2013, *L. infantum* was found in 2.24% of examined dogs [59]. In Montenegro, 66 cases of human VL and one case of CL were found in 2015 [60]. Twenty-two VL patients were diagnosed by microscopic examinations in an epidemiological and diagnostic study on VL from the period of 2001–2007 in Belgrade, Serbia. At least 12 of them had a recent travel history to neighboring countries including coastal regions in Montenegro, Bosnia and Herzegovina, Greece and Albania, suggesting that the causative agent is circulating in different Balkan countries [61]. *Leishmania* spp. was detected in 15 out of 206 spleen samples from golden jackal (*Canis aureus*) in the Vojvodina region, Serbia in 2014 [62]. Similar results between 2003 and 2016 on either parasite circulation between wild hosts and vectors in natural ecosystems [63, 64] or parasite detection in humans and stray dogs [65–68] were reported from nearby countries, such as Greece, Albania and Romania. These findings strongly suggest that the causative agents are currently circulating in the Balkans, presumably supported by the widespread presence of vectors as reported in this study. In addition, two recent studies put under the spotlight previously neglected sand fly-borne viruses, demonstrating the presence of two lineages of Toscana virus in Croatia in 2017 [69] and Balkan virus in Croatia and Bosnia and Herzegovina in 2017 [70]. All these findings clearly highlight that detailed determination of current sand fly presence and distribution in the Balkans is of utmost importance to understand the dynamics of sand fly-borne diseases in this region.

While historical records provided valuable information and faunistic lists on sand fly fauna in the study area, they have important limitations: (i) due to unavailability of most collected material, it is impossible to confirm their identity using morphological and molecular methods; (ii) studies performed in the same areas in consecutive years do not provide consistent results in terms of the species present; (iii) species were reported in certain areas which according to our results are far away from their natural distribution; (iv) since most of the studies were performed on sand fly fever and VL or CL epidemics, published species lists may not include species that were present but occurred in habitats with no pathogen transmission; and (v) there is an inconsistency in the literature in the use of some species names (e.g. *P. simici vs P. chinensis*). For these reasons, it seems relevant to compare the results of this study mostly with the faunistic data published recently.

The field study performed by our international team in 2014–2016 provides faunistic data obtained from large-scale field collections performed in the same seasons of three consecutive years with highly standardized sampling methods. The applied morphological species identification provided a reliable and comparable species list for each country in the study area. Moreover, storage of remaining material and reference to the slide-mounted vouchers enable use of molecular techniques (DNA barcoding and MALDI-TOF MS protein profiling) for confirmation of species identification in the future. Thus, limitations of previously published data on sand fly fauna from the region mentioned above were reduced and a clear profile of the sand fly fauna was obtained for at least eight countries. Moreover, this study presents the first integrated faunistic data for, at least, Montenegro, Bosnia and Herzegovina, Kosovo and North Macedonia. Analysis of the data enabled us to update species lists of some countries by removing some previous problematic identifications and adding the newly recorded species to the lists: two in North Macedonia, five in Bulgaria, three in Kosovo, two in Bosnia and Herzegovina and two in Montenegro (Tables 2, 3).

To date, few studies published on the sand fly fauna of Bosnia and Herzegovina reported *P. papatasi*, *P. major* (*s.l*.), *P. simici*, *P. tobbi* and *S. minuta* [30, 71]. Our study confirmed three of four species identified 70 years ago and added *P. neglectus* and *P. mascittii*. Previously reported *P. major* is probably *P. neglectus*, a species of the *P. major* (*s.l*.) complex. When the data are combined, it is recorded that there are six species of sand flies in Bosnia and Herzegovina.

Studies on the sand fly fauna of Kosovo are also very limited. The studies published in 1949 and 1974 showed the presence of *P. major*, *P. perfiliewi*, *P. simici*, *P. tobbi, P. sergenti*, *P. papatasi*, *P. balcanicus* and *P. neglectus* [25, 72]. The results of our study confirmed six species identified in previous studies, except *P. major* and *P. sergenti*, and also added three new records, *P. mascittii*, *P. alexandri* and *S. minuta*, to the species list.

Although studies performed during the former Yugoslavia period provided some information about the species composition in Montenegro, there was no complete, standardized and updated faunistic information for this country. Studies conducted between 1950 and 1995, especially in the Bar region, identified six sand fly species, namely *P. papatasi*, *P. perfiliewi*, *P. neglectus*, *P. tobbi*, *P. kandelakii* and *S. minuta* [26, 28, 34, 35, 73–77]. The results of our study confirmed the previously identified species *P. neglectus*, *P. tobbi* and *S. minuta*. However, *P. perfiliewi*, *P. papatasi*, *P. major* and *P. kandelakii* were not recorded in Montenegro while *P. simici* and *P. balcanicus* were added to the species list by the present study as new records. Absence of *P. papatasi* and *P. perfiliewi*, recorded in previous studies in Montenegro and recorded by this study in neighboring countries, may be attributed to the fact that Montenegro was the second country with the lowest number of sampling stations (*n* = 51) in our entire study area.

North Macedonia, neighbouring Greece, Bulgaria, Albania, Kosovo and Serbia, is a landlocked country located in the middle of the study area. Its geographical structure allows sand fly populations to be found at altitudes of 300–400 m, especially in mountain skirts. Indeed, North Macedonia was the country with the highest number of species (*n* = 11). In the studies conducted between 1926 and 1974, 10 sand fly species were recorded in the country [22, 25, 26, 49, 71, 73, 77]; however, no record on sand fly fauna has been published for this country in the past 46 years. In this respect, our study presents a first updated species list for North Macedonia after four decades. The analysis of the specimens we collected during our field studies confirmed the 10 species previously identified in the country. Two specimens of the subgenus *Paraphlebotomus*, could not be morphologically identified at the species level. In addition, *P. alexandri* was added to the new species list.

In this study, we collected sand fly specimens at 63 locations and 216 sampling stations in the central and southern regions of Bulgaria, where the VL, CanL and even CL cases have been historically recorded, and also in the Black Sea coast. We identified nine different sand fly species in this country. As detailed in the result section, only scarce notes on sand fly fauna of Bulgaria were previously published and within these, some misidentifications should be considered. We found only two articles summarizing the sand fly fauna of Bulgaria in 2013 and 2015 [46, 78]; however, none of these presented the results based on data from field studies. Hristova [79] reported in 2005 the presence of *P. chinensis* (misspelled as “*P. shimemsis*”) in the Thrace and Danube valleys, however, with no details regarding its occurrence. Considering the currently known distribution (East Asia) of *P. chinensis*, the species identification of Hristova [79] may be regarded as incorrect and possibly refers to historical use of this name for several taxa described later as distinct species within subgenus *Adlerius.* The first record named *P. chinensis* is given by Boychev in 1950 [47] who reports the species as abundant in the Vidin area. The study does not provide the decisive morphological characters needed for a conclusive species identification (ascoid formula, number of coxite hairs); however, the note on the size of the collected specimens excludes *P. simici*. While the drawing of a spermatheca suggests *P. balcanicus*, both the drawing and photo of male terminalia also resemble *P. longiductus*. We think that the material of *P. perniciosus* added to the Bulgarian sand fly list [42] and reported again in 1950 [47], was probably *P. tobbi*, considering the distribution of *P. perniciosus* in the western part of the Mediterranean. To conclude, of five sand fly species reliably recorded in Bulgaria in the past (*P. papatasi*, *P. sergenti*, *P. balcanicus*, *P. tobbi* and *S. minuta*), we confirmed the presence of all except *P. balcanicus* and added five new records to the species list: *P. alexandri*; *P. kandelakii*; *P. neglectus*; *P. perfiliewi* (*s.l*.); and *S. dentata*. Nevertheless, we consider the presence of *P. balcanicus* in Bulgaria very likely as it is present in the neighbouring countries, namely Turkey [80], Greece [31], North Macedonia, Serbia, Kosovo and Romania [81].

The sand fly fauna in Croatia is one of most studied in the former Yugoslavia. Previous studies focused on the Adriatic coastline and islands where cases of leishmaniasis and phlebovirus infections were recorded in the past. The presence of *P. neglectus* and *P. perniciosus* was recorded in 1931 [22]. Between 1936 and 1990, many researchers conducted extensive field studies in the country [23, 30, 36, 73–75, 82–84]. In recent years, Živičnjak et al. [85] in 2011 confirmed *P. papatasi*, *P. neglectus*, *P. tobbi* and *S. minuta* in the country. Ayhan et al. [69] reported the presence of *P. neglectus*, *P. tobbi* and *S. minuta* in Croatia in their sub-study within VectorNet Project. All these studies showed presence of 11 sand fly species in Croatia. In our study, *P. neglectus*, *P. perniciosus*, *P. papatasi*, *P. tobbi* and *S. minuta* were confirmed.

Among the countries of former Yugoslavia, special attention should be paid to Serbia. After the cases of leishmaniasis which reappeared there in 1968, it was considered as eradicated for several decades, until re-emerging again since 1999. Many cases of imported CanL have been identified in the Vojvodina region, the northern part of Serbia where the disease was not endemic previously [86], followed by autochthonous cases [87]. Simultaneously, first imported and subsequently autochthonous sporadic cases have been reported in the south and southeast of Serbia in the previously both endemic and non-endemic regions [61, 88]. As we mentioned above, numerous studies on sand fly fauna were previously performed by different entomologists also in Serbia. In 1998, Miscevic et al. [28] presented a collective list of the identified species for Serbia in a review. They showed a total of eight species, i.e. *P. papatasi*, *P. perfiliewi*, *P. neglectus*, *P. tobbi*, *P. simici*, *P. sergenti*, *P. balcanicus* and *S. minuta.* Our studies confirmed all species and added two new records to the species list: *P. mascittii* and *P. alexandri.*

In Slovenia, where we detected the lowest number of sand fly species (4 species) in our study area, the first published record of sand flies in the country is from 2015 [37]. Our study was carried out with 48 sampling sites in the Istrian Peninsula and confirmed the presence of *P. neglectus*, *P. perniciosus*, *P. papatasi* and *P. mascittii*. Ivovic et al. [37] noted that *S. minuta* is also abundant in Slovenia [37].

The literature review showed that 12 sand fly species have been previously identified within the entire study area. However, the report of *P. major* can probably be regarded as misidentification of *P. neglectus*. Within the subgenus *Larroussius*, *P. major* (*s.l*.), also referred to as “major group”, represents a species complex that comprises six genetically distinct species with similar morphology [89, 90]. Within this complex, *P. major* is expected to occur in India, Nepal and Pakistan; we may therefore assume that the historically recorded *P. major* specimens were in fact *P. neglectus* that is the only species of the complex occurring in Europe. The same occurs with the single *P. syriacus* record in the study area [1], another species within the *P. major* group that is expected to be distributed in the Middle East and shows morphological characters very close to *P. neglectus*. Within the subgenus *Larroussius*, a single record of *P. pedifer* [32] also added to the species list of former Yugoslavia. However, it was not confirmed by any later survey until now. The original study [32] does not provide details of decisive morphological characters used for the species identification and no graphical material that would support this identification or vouchers for re-examination are available. As *P. pedifer* is only known from Kenya, Sudan and Ethiopia [31], we consider the record in the Balkan region as highly unlikely and most probably a misidentification.

Our field study revealed a total of 14 species in the study area. All predominant sand fly species found belong to subgenus *Larroussius* including predominant species *P. neglectus*, *P. perfiliewi* and *P. tobbi*. In addition, two other *Larroussius* species were found, *P. perniciosus* in Croatia and Slovenia and *P. kandelakii* in Bulgaria. All these sand fly species are proven vectors of *L. infantum* causing zoonotic VL and human VL. Moreover, *P. perniciosus* and *P. tobbi* were recently found permissive for *L. tropica* [91, 92]. This parasite is the causative agent of anthroponotic and zoonotic CL which in Europe sporadically occurs in Crete [93] but is also frequent in refugees from Middle East and Central Asia arriving in Europe [94].

The subgenus *Paraphlebotomus* was represented by *P. sergenti* and *P. similis*, proven or suspected vectors of *L. tropica* while a single European member of subgenus *Phlebotomus*, *P. papatasi*, is widely known as vector of *L. major* causing zoonotic CL in Middle East and Maghreb area. In Europe, however, no suitable reservoirs for *L. major* were identified; therefore, the spread of this disease, in contrast to *L. tropica*, is highly unlikely in the Balkan region. The data obtained from our field study therefore represent a valuable material for preparation of probability and risk maps/analysis of leishmaniasis for each country.

## Conclusions

The correct identification of the sand fly species provides a better understanding of the sand fly-pathogen relationship as well as the planning of ecological studies on populations, the sensitivity of risk analysis on sand fly-borne diseases and ultimately for the success of disease control measures. In this study, the presence of 14 sand fly species of two genera was recorded in eight Balkan countries by a large-scale field survey that thoroughly updated the knowledge of sand fly fauna in this important and yet understudied region. For the first time, a sand fly species checklist was prepared for four countries and an update of sand fly fauna was provided for the remaining surveyed countries with comparison of previously published and often much outdated data after at least three decades. Critical assessment of both historical and recent data confirmed the presence of 14 species and showed that species lists have changed over time for each country by both more detailed sampling and new insights into sand fly taxonomy. In all surveyed countries, proven or suspected vectors of *L. infantum* were found, with omnipresent *P. neglectus* and both *P. tobbi* and *P. perfiliewi* in most countries We believe that our findings will contribute to future research focused on sand fly-borne diseases in the Balkans and also in the rest of Europe.

## Supplementary information

**Additional file 1: Table S1.** Historical data review on the sand fly fauna of the study area. Table summarizing essential published literature between 1910 and 2019.

## Data Availability

All data generated or analyzed during this study are included in this published article and its additional files. Sand fly collections are stored in vials with 70% ethanol in -80 °C freezer or as DNA extraction materials in vials in -80 °C freezer or as prepared slides in collection boxes and are available for consultation at HU-VERG (Hacettepe University, Faculty of Science, Biology Department, Ecology Section, Vector Ecology Research Laboratories, Beytepe-Ankara, Turkey) and at CUNI (Charles University, Faculty of Science, Department of Parasitology, Praque, Czech Republic) and at UPBIO (University of Primorska, Biodiversity Sciences and Informatics, Koper, Slovenia) and at the University of Novi Sad, Faculty of Agriculture, Department of Phytomedicine and Plant Protection, Laboratory for Medical Entomology, Novi Sad, Serbia and at Unite des Virus Emergents (UVE: Aix Marseille Univ, IRD 190, INSERM 1207, IHU Mediterranee Infection), Marseille, France. Raw data on sand fly fauna, species distribution, geocoordinates of sampling stations, physical properties of sampling locations were delivered to ECDC and EFSA.

## Abbreviations

m.a.s.l: metres above sea level
CL: cutaneous leishmaniasis
CanL: canine leishmaniasis
ECDC: European Centre for Diseases Prevention and Control
EFSA: European Food Safety Authority
MALDI-TOF MS: matrix assisted laser desorption/ionization-time of flight mass spectrometry
PAST: palaeontological statistics
VBORNET: European Network for Arthropod Vector Surveillance for Human Public Health
VECTORNET: An European network for gathering and sharing data on the geographic distribution of arthropod vectors of disease agents affecting humans and livestock
VL: visceral leishmaniasis
WHO: World Health Organization
