# Supplementary material for: Sand flies (Diptera: Psychodidae) in eight Balkan countries: historical review and region-wide entomological survey
Source: Parasit Vectors. 2020 Nov 11;13:573. doi: 10.1186/s13071-020-04448-w (PMC7661266; doi:10.1186/s13071-020-04448-w)
Supplement: Supplementary file 1 — Additional file 1: Table S1. Historical data review on the sand fly fauna of the study area. Table summarizing essential published literature between 1910 and 2019. [file 13071_2020_4448_MOESM1_ESM.docx]

| Additional file 1: Table S1. Historical data review on the sand fly fauna of the study area. Table summarizing essential published literature between1910 and 2019 | | | | | | | |  |
| --- | --- | --- | --- | --- | --- | --- | --- | --- |
|  | | |  | |  |  |  |  |
| **Author(s)** | **Date** | **Country** | | **City / Locality** | | | **Species** | Ref.No. |
| Nedyalkov N | 1909 | Bulgaria | | Stara Zagora | | | *S.minuta* | [53] |
| Nedyalkov N | 1912 | Bulgaria | | Plovdiv, Sliven | | | *S.minuta* | [54] |
| Shishkov G, Konsulov S | 1914 | Bulgaria | | Ruse, Sliven, Bachkovo | | | *P.papatasi* | [55] |
| Drenski K. | 1926 | Bulgaria and North Macedonia | | N/A | | | *P.papatasi* | [49] |
| Drenski P, Drenski K | 1928 | Bulgaria | | Varna, Plovdiv, Stara Zagora, Nova Zagora, Elhovo, Burgas, Sozopol, Sliven | | | *P.papatasi, P.sergenti, P.perniciosus S.minuta* | [42] |
| Adler S, Theodor O | 1931 | Croatia, North Macedonia Yugoslavia | | N/A | | | *P.neglectus, P.perniciosus P.perfiliewi, P.major* | [22] |
| Drenski P | 1931 | Bulgaria | | Levunovo | | | *P.papatasi* | [50] |
| Tartaglia P | 1936 | Croatia | | Dalmatia | | | *P.neglectus, P.tobbi* | [23] |
| Drenski P | 1942 | Bulgaria | | Varna | | | *P.papatasi* | [51] |
| Simić Č, Živković V | 1949 | North Macedonia | | Skopje, Kumanovo, Kriva Palanka  Ovce polje, Omorane | | | *P. simici, P.neglectus, P.perfiliewi  P.papatasi, P.sergenti, P.tobbi S. minuta, S.dentata* | [25] |
| Simić Č, Živković V | 1949 | Western Yugoslavia Kosovo, Vojvodina | | Presevo, Pcinja, Leskovac, Nis Aleksinac, Dobric, Poljanica, Moravica Kacanik, Donji Livac, Giljane, Gracanica Pristina, Prizren, Orahovac, Dakovica | | | *P.major, P.papatasi, P.perfiliewi  P.simici, P.tobbi, P.sergenti* | [25] |
| Ganov V | 1949 | Bulgaria | | Pleven, Veliko Tarnovo, Balchik | | | *P.papatasi* | [48] |
| Živković V | 1950 | Serbia | | Vranje, Leskovac, Prokuplje  Nis, Aleksinac | | | *P.major, P.papatasi, P.perfiliewi P.sergenti, P.simici, P.tobbi* | [26] |
| Simić C et al. | 1950 | Yugoslavia, Montenegro | | Kotor, Przno, Rezevic, Bijela, Kumbor Sutorina, Bioc, Zabalj, Cetinje | | | *P.major,P. tobbi, P.papatasi S.minuta* | [73] |
| Simić C | 1950 | North Macedonia, Montenegro Croatia | | Stari grad, Omoran, Titograd, Kotor Novi Bar, Crnojevića Reka, Komarin, Solin, Split, Stobređe, Klisa, Kaštel  Brač, Vrpolje, Šibenik, Benkovac, Vrani, Sukošan | | | *S. dentata, S.minuta* | [26] |
| Simić C et al. | 1950 | Croatia | | Metković, Dragljane, Solin Split, Blato, Biograd na Moru, Ploče | | | *P. major, papatasi, tobbi perfiliewi, S.minuta* | [73] |
| Boychev D. | 1950 | Bulgaria | | Vidin, Novo selo, Vrav, Gamzovo, Pleven, Mahalata, Gorni Dabnik, Dolni Dabnik, Yasen, Lom, Oryahovo, Lukovit, Dermantsi | | | *P.chinensis, P.papatasi, P.sergenti  P.perniciosus, S.minuta* | [47] |
| Simić C | 1951 | Bosnia&Herzegovina, Croatia Montenegro, Serbia  North Macedonia | | Dalmatia, Vojvodina Central-North West Serbia | | | *P.major, P.papatasi  P.perfiliewi, P.sergenti* | [71] |
| Simić C et al. | 1951 | Serbia, Bosnia & Herzegovina Croatia | | Vojvodina region, Višegrad, Goražde Mostar, Čapljina, Vitina, Bijeli Breg  Ilijica, Bkšum, Deržnica, Ljubušk Split, Solin, Trogir, Benkovac, Zadar Opatija, Pula, Rovinj, Zagreb | | | *P. papatasi, P.perfiliewi, P.simici P.major, P.papatasi, P.tobbi, S.minuta* | [30] |
| Drenski P. | 1955 | Bulgaria | | Dulovo | | | *P.papatasi* | [52] |
| Tartaglia P | 1957 | Croatia | | Dalmatia | | | *P.papatasi, P.perfiliewi* | [23] |
| Živković V | 1969 | Serbia | | Leskovac, Central, west and east Serbia | | | *P.major, P.perfiliewi, P.papatasi P.sergenti, P.simici, P.tobbi* | [76] |
| Bordoski A, Savin Z | 1970 | Montenegro, Croatia | | Bar, Makarska, Omis, Split | | | *P.papatasi, P.neglectus, P.perfiliewi  tobbi, S.minuta* | [74] |
| Živković V, Miščević Z | 1970 | Croatia, Montenegro | | Dalmatia, Adriatic Coast | | | *P.papatasi, P. neglectus, P.perfiliewi P.tobbi, S.minuta* | [75] |
| Živković V, Miščević Z | 1972 | Serbia | | Nis, Merošina, Brest | | | *P.papatasi, P.major, P.sergenti,P.perfiliewi P.tobbi, P.simici, P.balcanicus* | [29] |
| Živković V et al. | 1973 | North Macedonia | | Skopje, Kumanova, Titov Veles, Kumanovo  Skopje, Novo Selo, Rakotinci, Dobrošane  Šuplji kamen, Pcinja, Stari Grad | | | *P. papatasi, P.perfiliewi, P.major, P.tobbi,  P.simici (chinensis s.l.), S. minuta, S.dentata* | [77] |
| Živković V | 1974 | North Macedonia, Kosovo | | Skopje, Matka, Aračinovo, Konopnica Psača, Gradac, Kriva Palanka Barje Čiflik, Gnjilan | | | *P. balcanicus* | [72] |
| Živković V | 1974 | Serbia | | Dobric | | | *P.major, P.perfiliewi* | [72] |
| Živković V | 1980 | former Yugoslavia | | Croatia, North Macedonia, Serbia Northern Dalmatia, Istra Peninsula | | | *P.papatasi, P.sergenti, P.neglectus P.perfiliewi, P.perniciosus, P.tobbi, P.simici P.balcanicus, S.minuta, S.dentata* | [21] |
| Gligić A et al. | 1982 | Serbia | | Dobric-Merosina, Jug, Bogdanovac  Aleksandrovo, Oblacina, Krusce | | | *P.papatasi,P. perfiliewi P.major, P.simici* | [19] |
| Živković V | 1982 | Serbia | | Aleksinac | | | *P.major, P.perfiliewi, P.simici P.tobbi, P.papatasi* | [24] |
| Živković V | 1982 | former Yugoslavia | | Serbia, North Macedonia Croatia, Bosnia&Herzegovina | | | *P.perfiliewi* | [24] |
| Živković V | 1982 | Serbia | | Dobric, Jug Bogdanovic | | | *P.sergenti, P.major, P.perfiliewi P.tobbi, P.simici* | [24] |
| Živković V | 1982 | Serbia | | Sout-east Serbia, Dobric | | | *P.papatasi, P.perfiliewi,P.neglectus P.simici, P.tobbi, P.sergenti, P.balcanicus* | [24] |
| Additional file 1: Table S1. Historical data review on the sand fly fauna of the study area. Table summarizing essential published literature between 1910 and 2019 (Continued) | | | | | | | |  |
| **Author(s)** | **Date** | **Country** | | **City / Locality** | | | **Species** | Ref.No. |
| Borcić B et al. | 1990 | Croatia | | Dalmatia-Korcula, Hvar, Brac Islands | | | *P.papatasi, P.neglectus, P.tobbi, S.minuta* | [83] |
| Bisevać et al. | 1990 | Croatia | | Mljet Island | | | *P.papatasi, P.perfiliewi, P.sergenti  P.tobbi, P.perniciosus, S.minuta* | [84] |
| Wagner R | 1990 | Bulgaria | | N/A | | | *P.balcanicus* | [43] |
| Technical Report (WHO) | 1990 | former Yugoslavia | | N/A | | | *P.neglectus, P.perfiliewi, P.simici P.tobbi, P.sergenti* | [2] |
| Seccombe AK et al. | 1993 | Bulgaria | | N/A | | | *P. simici* | [31] |
| Seccombe AK et al. | 1993 | former Yugoslavia | | N/A | | | *P.balcanicus, P.perfiliewi, P.tobbi P.sergenti, P.mascittii, S.minuta* | [31] |
| Anonymous (AFPB-US) | 1993 | Yugoslav Republics | | Serbia-Vojvodina, Kosovo-, Croatia,  Bosnia&Herzegovina, Slovenia, Macedonia Montenegro | | | *P.balcanicus, P.major, P.mascittii P.papatasi, P.pedifer, P.perfiliewi P.perniciosus, P.sergenti, P.simici P.similis, S. dentata, S.minuta* | [32] |
| Milutinović M et al. | 1995 | Montenegro | | Ulcinj | | | *P. tobbi, P.major, S.minuta* | [33] |
| Miščević Z et al. | 1998 | Yugoslavia, Serbia Montenegro, Croatia | | East and South-east, Bar, Coastal region Mljet island, Blato, Kozarica, Babino polje, Korita | | | *P.papatasi, P.sergenti (similis), P.neglectus P.perfiliewi, P.perniciosus, P.tobbi, P.simici P.balcanicus, S.minuta, S.dentata* | [28] |
| Ivović V et al. | 2003 | Montenegro (Yugoslavia) | | Bar area | | | *P.neglectus, P.tobbi, P.papatasi P.perfiliewi, S.minuta* | [34] |
| Ivović V et al. | 2004 | Montenegro | | Bar area | | | *P.neglectus, P.tobbi, P.perfiliewi P.papatasi, P.kandelakii, S.minuta* | [35] |
| Hristova T. | 2005 | Bulgaria | | Trace Valley, Danube Valley | | | *P.papatasi, P.chinensis* | [79] |
| Bosnić S et al. | 2006 | Croatia | | Sibenik-Knin, Split-Dalmatia, Dubrovnik-Neretva | | | *P.tobbi, P.neglectus, P.perfiliewi P.mascittii, S. minuta* | [36] |
| Ready PD | 2010 | former Yugoslavia | | Serbia, North Macedonia, Montenegro, Bosnia and Herzegovina, Croatia, Slovenia | | | *P.sergenti,P. perfiliewi, P.neglectus P.tobbi, P.papatasi* | [95] |
| Živičnjak et al. | 2011 | Croatia | | Sibenik-Knin, Split-Dalmatia | | | *P. neglectus, P. tobbi, P. papatasi  S. minuta* | [85] |
| Radev V et al. | 2011 | Bulgaria | | Smolyan, Yambol, Kardzhali, Haskovo, Blagoevgrad | | | *P.tobbi, P.papatasi, P.sergenti* | [44] |
| Harizanov et al. | 2013 | Bulgaria | | N/A | | | *P. papatasi, P. sergenti, P. balcanicus P. tobbi* | [78] |
| Maroli M et al. | 2013 | Croatia, Kosovo, North Macedonia, Montenegro, Romania, Slovenia | | N/A | | | *P. neglectus, P.perfiliewi* | [1] |
| Mikov O et al. | 2015 | Bulgaria | | N/A | | | *P.balcanicus, P. papatasi, P. perniciosus  P . similis, P. tobbi* | [45] |
| Pantchev N et al. | 2015 | Bulgaria | | N/A | | | *P.tobbi* | [46] |
| Ivović V et al. | 2015 | Slovenia | | Istrian Peninsula | | | *P.neglectus, P.perniciosus, P.papatasi P.mascittii, S.minuta* | [37] |
| Vaselek S et al. | 2017 | Serbia | | Vojvodina | | | *P.papatasi, P.perfiliewi,  P.neglectus, P.mascittii* | [38] |
| Vaselek S et al. | 2019 | Serbia | | immigrant routes, shelter/camps tourist/trade transit routes | | | *P.papatasi,P. perfiliewi, P.tobbi, P.neglectus P. sergenti, P.alexandri, P.simici P.balcanicus, P.mascittii* | [27] |
